# Dust Emission Monitoring in Cement Plant Mills: A Case Study in Romania

**DOI:** 10.3390/ijerph18179096

**Published:** 2021-08-28

**Authors:** Cristian Ciobanu, Irina Aura Istrate, Paula Tudor, Gheorghe Voicu

**Affiliations:** 1 Department of Biotechnical System, Faculty of Biotechnical Systems Engineering, University Politehnica of Bucharest, Splaiul Independentei 313, 060042 Bucharest, Romania; ciobanu_77@yahoo.com (C.C.); ghvoicu_2005@yahoo.com (G.V.); 2 Ceprocim Sa, Strada Preciziei 6, 062203 Bucharest, Romania; 3 Department of Management, Faculty of Entrepreneurship Business Engineering and Management, University Politehnica of Bucharest, Splaiul Independentei 313, 060042 Bucharest, Romania

**Keywords:** cement industry, PM, dust emissions, cement factories

## Abstract

This paper presents aspects of monitoring material dust emissions from stationary emission sources (monthly dust measurements performed on cement mill stacks—mill outlet and separator outlet). Additionally, the Portland cement mill technological process (its component parts), as well as the solutions regarding the reduction of the air emissions level, following the emission limit values (VLE), established in the integrated environmental authorization (AIM) from a cement factory in Romania, were analyzed. The paper focused on analyzing the data obtained in three different years for PM10 and dust concentrations (2018–2020). For each year, the measurements have been done in 3 months, each in a different season. The average values for each year for working conditions were: 30.22 mg/m^3^ (2018), 27.38 mg/m^3^ (2019), and 27.51 mg/m^3^ (2020) for working conditions and for normal conditions: 34.22 mg/m^3^ (2018), 30.49 mg/m^3^ (2019), and 30.16 mg/m^3^ (2020). For all 3 years, the values measured in spring were higher than the other two, both for work and normal conditions.

## 1. Introduction

Air pollution is a problem of particular importance and directly affects the quality of human life and the proper conduct of human activities. Moreover, the almost aggressive industrialization that has occurred in recent decades has visibly affected the quality of the air we breathe, a significant part of the world’s population suffering from pollution with various harmful substances and suspensions of material particles in the air.

One important element that must be identified when we analyze the impact of a cement plant is the air quality index (AQI). It is provided by measuring stations, which provide information for its calculation, comprising the main pollutants (total particulate matter with PM_10_—particles with dimensions less than 10 µm), to which the auxiliary pollutants are added (PM_2.5_—included in PM_10_). These stations make measurements at fixed intervals of 15 min based on which hourly averages are established, and the air quality index is calculated [1]. The air quality index is given by the highest value of the pollutants included in the determination. The conversion is made from the measurement unit transmitted by the ppm sensors (parts per million) to μg/m^3^. Regarding the particles emitted into the atmosphere, PM_10_ and PM_2.5_, for the values below 15 μg/m^3^ (PM_10_), and 10 μg/m^3^ (PM_2.5_) the pollution level is set as very low, while for values higher than 100 μg/m^3^(PM_10_), and 60 μg/m^3^ (PM_2.5_), the pollution level is very high.

Historically, in terms of dust emissions, these have been the main concerns related to the cement manufacture anywhere in the world. In the cement industry, dust is emitted from various processes, such as handling raw materials, crushing of limestone, kiln burning, clinker production and storage, cement finishing, and power utilities (the coal mill and the power generators) [2,3].

Recent improvements in cement production processes and pollution control technologies have reduced pollutant emissions into the atmosphere. However, even with the best methods, the amount of pollutants released depends on the cement produced and the type and quality of fuel burned in limestone kilns, especially when it contains a large amount of waste.

Among the substances that cause air pollution, particulate matter (PM) is seen by some studies as the most harmful. PM can have various health effects, such as asthma, bronchitis, cardiovascular disease, and lung cancer [4].

Particulate matter (PM) is not a single pollutant; instead, it is a mixture of many chemical elements, namely a complex mixture of solid particles and aerosols consisting of tiny droplets of liquids, dry solid fragments, and solid particles coated with liquid. The particles vary significantly in size, shape, and chemical composition and may contain inorganic ions, metal compounds, elemental carbon, organic compounds, and compounds in the earth’s crust. Particles with a diameter less than or equal to 10 microns (PM_10_) are inhalable in the lungs and can induce various adverse health effects.

Particles with dimensions finer than 2.5 microns (PM_2.5_) can have the same source or a different source compared to PM_10_, so are different chemical compositions. However, in most locations in Europe where there are cement plants, it is estimated that PM_2.5_ constitutes 50–70% of PM_10_ [5].

Rovira et al. [6] found that PM_10_ in cement powder consists of mineral matter, sea spray, secondary inorganic aerosols, organic matter plus elemental carbon, trace elements, or indeterminate fraction, making determinations on six consecutive days in two distinct periods of the year (cold season, hot season). In addition, they found no differences between working days and weekends in PM_10_ concentrations (respectively in their composition—Al, Ca, Ni, V), but found that there are differences in the sampling period (cold season or warm season).

We have seen that humans can be negatively affected by exposure to ambient air pollutants. For this reason, the EU has developed many standards that set allowable levels for various contaminants that may occur in the environment, including small particulate matter. They apply over different periods, as the observed health impact associated with multiple pollutants occurs at different exposure periods. Thus, for PM_2.5_, the average permissible concentration for 24 h, not exceeding 35 times for 1 year, is 25 μg/m^3^, while for PM_10_, it is 35 μg/m^3^ for the same exposure period, according to Directive 2008/50/EC [7], while the indicative value given by the World Health Organization (WHO) for “total suspended particles” is 120 μg/m^3^ [8]. For example, six specific intervals correspond to the concentrations of suspended particles less than 2.5 µm (PM_2.5_) in Romania. The arithmetic mean of the hourly values, recorded in the last 24 h, is included within one of the concentration ranges of 0–10; 10–20; 20–25; 25–50; 50–75, and 75–800 µg/m^3^, and an index from 1 to 6 is associated with each interval [9].

It was found that the finer the PM, the more harmful they are. PM’s size and chemical composition depend on several factors, such as weather, time of year, and source of emission [10]. The presence of toxic substances in PM (such as acids, metals, organic particles, and hazardous air pollutants—HAPs) has a negative impact on human and animal health, but also on their physical properties (size, number of particles, and specific surface area) [10,11,12,13,14].

Material particles finer than 2.5 microns were found in the atmosphere of urban areas, especially in winter, so the exposure to the environment and the risks to human health are higher in winter. Exposures are also more intense for indoor activities, where PM_1_ penetrates much more quickly than PM_10_. It is also found that indoor concentrations are about 65% of outdoor concentrations for PM_2.5–1_ and PM_1_ 45% of outdoor PM_10_ concentrations [13].

In the cement industry, a general feature of the stages for the process of venting the technological gas (air) into the atmosphere is that the exhaust gas passes through the pulverized material, resulting in a dispersion of gas and particles, which would make it easier to find primary measures reducing emissions. The particles generated are related to the source material, i.e., the raw materials (partially calcined), clinker, or cement.

It is well known that dust deposits, regardless of the area of origin, are smaller when the distance is greater from the source of dust, and the impact on the environment (flora and fauna) decreases with increasing distance. Thus, if the mass of particles deposited in the area of a cement plant was 24–83 mg/m^2^ per day (at source), it had much lower values of about 13–17 mg/m^2^ per day, at a distance of 300–800 m from the dust source [14].

It was also found that the dust deposited on the surfaces inside the cement factories in Benin could vary in the ranges of 0.96–9.36 g/m^2^/day and 8.87–18.96 g/m^2^/day at various points near the factories, depending on the month of the year. However, these values were higher than the international safety values (1 g/m^2^/day AFNOR and 350 mg/m^2^/day TA-Luft) and present dangers to human and animal health [15].

Addo et al. [16] also found that the total values of dust in the vicinity of a cement plant in Ghana were in the range of 217.86–1771.13 g/m^3^, with average values of 506.77 g/m^3^, with deviations depending on the direction of the prevailing winds [16]. Exposure to 200 µg/m^3^ of material particles for a long time can cause diseases of the upper respiratory tract in adults, and if it is in a range of 294–470 µg/m^3^, it affects the immune function in children. The study shows that the health effects of suspended dust particles depend on the particle size, concentration, and exposure time. Experimental research on rats shows that cement dust is pathogenic and toxic. Undoubtedly, people working in cement factories are at risk of being affected by various diseases that arise from exposure to cement dust. This requires using appropriate equipment and technologies to reduce the amount of cement dust that reaches the outside environment [17,18].

Most studies conducted worldwide state that cement plants emit many pollutants into the atmosphere, recognizing cement production as the largest source of PM emissions, accounting for up to 40% of total industrial emissions and between 25–30% of total PM emissions. An increase in production or a change in the type of fuel or dust control technology affects the volume and concentration of contaminants released [19,20,21].

The absence or presence of dust control technology affects the amount of dust emitted into the atmosphere. If no dust control technology is used, the percentage of PM can reach 24% for PM_10_ and about 7% for PM_2.5_, in the case of wet material processing, respectively, 40% and 18% for dry processing, for PM less than 10 μm and 2.5 μm. The application of the dust control system results in up to 85% fewer particles released into the atmosphere with a diameter below 10 μm, both for the wet and dry [19]. However, bag filters in the dry process show that 45% of the escaped particles are smaller than 2.5 μm, which means larger particles have already been retained. Using bag filters (BF) can successfully reduce PM emissions, with various studies indicating their effectiveness. Bag filters allow a higher inlet temperature of the gas-dust mixture and have a higher dust removal efficiency than electrostatic precipitators (EFs). An EF system requires a lower gas inlet temperature to effectively remove dust, which requires a more significant amount of cooling water in the gas conditioning tower to cool it from about 400 °C to about 100 °C. Moreover, a BF system can significantly reduce dust emissions while reducing CO_2_ emissions by saving electricity consumption [22].

Different associations have been observed between cement dust and various types of cancer, mortality, bone disease, kidney disease, respiratory disease, or cardiovascular disease. The risk of cancer by inhalation or aspiration of cement dust is very high, especially for people living near cement factories. Silica dust causes silicosis and severe lung disease, and the presence of chromium compounds in cement dust can lead to cancer. The severity of the conditions depends on the duration of exposure, the concentration and constituent of the dust, and the individual sensitivity, but no researcher rules out the possibility of disease. Long-term effects exist, especially in children born and raised near cement plants [18,19,23]. Workers in the cement industry (exposed to a total dust concentration of 10,180 µg/m^3^ and PM_10_ of 8049 µg/m^3^) presented a forced expiratory volume in one second (FEV1) and a forced mid-expiratory flow (FEF) of 25–75%, which were much smaller and had higher fully massive four-loop tadpoles (FMFT) compared to workers in other fields of activity (exposed only to 192 µg/m^3^ and PM_10_—177 µg/m^3^) [24]. Moreover, FEV1 decreases with dust exposure increasing, and the prevalence of respiratory symptoms is higher for these workers. In the vicinity of cement plants, relatively high concentrations of PM could be observed, reaching an average value of about 388 µg/m^3^ for PM_10_ and 386 µg/m^3^ for PM_2.5_. Still, at the same time, high instantaneous measurements were recorded up to 12,200 µg/m^3^ (PM_2.5_) [25]. The analysis results show that the number of diseases is higher as the dust particles are smaller (even below 1 mm). This way, the respiratory system has more to suffer than in the case of larger particles [26].

Exposure to dust of the cement plant workers and the inhabitants around them can cause significant discomfort and associated respiratory diseases. Thus, for example, the vital capacity and forced expiratory volume in one second percent were significantly lower for workers in a cement plant in Nigeria than those in other less exposed sectors [27].

Zeleke et al. [28] found that the highest dust exposure for workers in cement factories is in the section of a raw materials crusher (38.6 mg/m^3^), followed by the section of packaging (18.5 mg/m^3^) and protection (0.4 mg/m^3^). At the same time, the highest prevalence of respiratory symptoms for workers with high exposure was a stuffy nose (85%), followed by difficulty breathing (47%) and “sneezing” (45%).

In northern Italy (1.2 km), there were clear links between PM_10_ levels and absenteeism near a cement plant, generally 2 days after higher concentrations. An average, concentration increase of 10 μg/m^3^ of PM_10_ in the previous days was associated with a statistically significant increase of 2.5% (95% CI: 1.1–4.0%) in the rate of school absences. The average concentration of PM_10_ during the trial period was 34 μg/m^3^ (range 4–183 μg/m^3^). The trial was done for 3 school years from 2007–2010, respectively, for 541 school days and with 462 children on average [29].

Various studies have shown that flora and fauna are also affected. For example, an atmosphere laden with cement dust can affect forests and tree growth, and cement dust emissions have a negative effect on their radial growth, especially for pines [30,31,32]. The results for plants range from their development to crop productivity. These effects are decreased light for photosynthesis, increased leaf temperature and mineral availability, altered plant enzymes, and reduced leaf size, number, and leaf area [33,34].

An important tool to manage raw materials for cement production in any cement plant is the raw mill. Here, the raw materials are brought to the desired dimensions and then placed in the oven. During the size reduction process, dust emissions are managed by air pollution control equipment, bag filters, or electrostatic filters. Depending on this equipment’s efficiency, dust emissions from raw material mills can be a cause for concern in the cement plant [19,21]. Additionally, the grinding of cement in the cement mill/mills generates an appreciable amount of dust. It has been established that about 7–10% of the cement can be lost due to uncontrolled emissions in the cement mill [21]. Some studies show that 4–5% of dust emissions are due to the furnace’s supply, while other dust emission sources are crushers, clinker coolers, grinding, and material handling equipment [35].

Dust emissions from the cement industry have been significantly reduced over the last 20 years. The latest cutting-edge techniques (electrostatic precipitators or bag filters) have led to insignificant stack emissions if the cement plant is properly managed. Dust collection devices are effective if chosen, following the dust’s physical characteristics, such as total charge of the air-dust mixture, particle size distribution, bulk density, electrical resistivity, and the volume of transport or suction gas. These devices are integrated into a dust collection and control system, consisting of centrifugal separators (cyclones and cyclone batteries) and specific filters (bag filters or electrostatic filters). Cyclones are used for coarse mixtures of air-material particles, while filters retain much smaller dust particles, placed in the technological flow after the cyclone batteries. The restriction of industrial emissions, together with continuous monitoring and appropriate regulations, is necessary to ensure that the levels of PM (airborne particles) in the ambient air are maintained at the recommended levels to protect the respiratory health of nearby community residents [36].

In Romania, there are several cement factories with different locations. In the technological flow of cement manufacturing, there are cement ball mills, combustion furnaces, and reduction and control systems for pollutant emissions into the atmosphere. Our paper presents determinations of dust emissions (dust concentration in dry gaseous conditions—mg/Nm^3^) at the two exhaust stacks of the ball mill assembly, namely the stack after the bag filter at the outlet of the cement mill and the stack after the bag filter at the outlet of the mill separator (as they are presented in Materials and Method). Moreover, the investigations regarding these emissions are presented, the measurements being performed monthly for 2 years at a cement factory in Romania.

## 2. Materials and Methods

**Figure 1:**
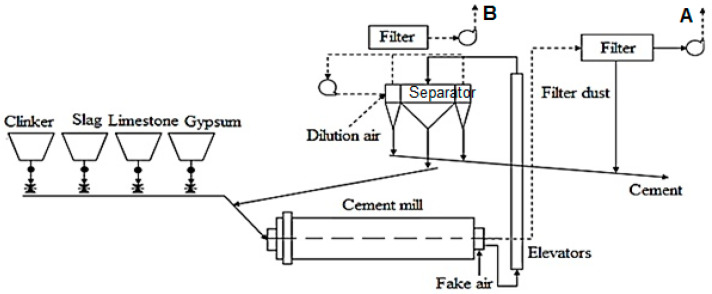
The flowchart of cement grinding, (**A**,**B**)—sampling points (evacuation stacks) [37].

The cement factory where the measurements were made was located in Romania’s center-north and had a final section with two cement mills. Cement grinding was performed inside the cement mill workshop. This manufacturing phase’s product was cement—a powdery, finely ground material in closed-loop tubular ball mills (Figure 1). The monthly amount of cement produced can be over 100,000 tons. Depending on the type of cement required, the materials introduced for grinding were:

- Clinker and plaster (addition of setting regulator) for type I cement (Portland cement with high initial strength for special structures);
- Clinker, gypsum, and hydraulic additives (slag, ash, limestone) for type II cement (Portland cement with slag with high initial strength).

The factory has been modernized by introducing specific dedusting equipment used in the production, transport, and storage processes. The installation is equipped with specific filters (bag filters or electrostatic filters). This has reduced the amount of dust released into the atmosphere and flue gas emissions, which are now well below the legal limits. The main dedusting machine is a state-of-the-art bag filter, which guarantees a maximum emission of 10 mg/Nm^3^, considered the most advanced equipment for dust retention used in the cement industry.

Bag filters are compact textile filters with a system of total self-dusting of the filter bags by compressed air jets. They are used for the dry separation of dust particles or the recovery of useful dust from air and gas currents. Textile filters can be used at temperatures up to 250 °C.

For bag filters, it is necessary to periodically clean the filter medium to control the gas pressure reduction as it passes through the filter. The bag filter has several compartments that can be individually insulated in the event of a bag failure. There are enough compartments to allow proper performance to be maintained if a compartment does not work. There are bag break detectors in each compartment to indicate the need for a maintenance operation if this occurs.

**Figure 2:**
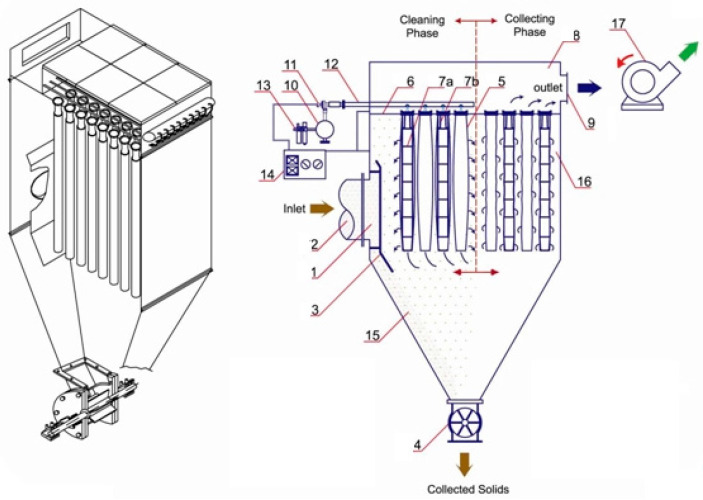
Bag filter example: 1. Separation chamber; 2. Dust laden gas inlet; 3. Baffle plate; 4. Rotary valve; 5. Filter bags; 6. Cell plate; 7. Filter Cage and Venturi; 8. Clean gas chamber; 9. Clean gas outlet; 10. Compressed air tank; 11. Diaphragm valve; 12. Blow pipe, 13. Water trap; 14. Sequential controller; 15. Falling dust; 16. Filtration chamber; 17. Suction fan [38].

An example of a bag filter is shown schematically in Figure 2.

Returning to the cement plant for which the analysis is made, the granulated blast furnace slag is dried in a rotary dryer using hot air recovered from the grate and/or natural gas cooler from the auxiliary hearth in a fluidized bed dryer with natural gas. The gypsum transported by car to the factory is discharged to a conical receiving hopper. It is taken over by a metal plate conveyor, a conveyor belt, and a chain elevator. The plaster is then taken over by a system of conveyor belts and stored in the mill silo. The clinker, gypsum, and grinding additives are extracted from silos, dosed, homogenized, and fed into cement mills. Cement mills are tubular ball mills, bicameral, and operate on a closed process. The mill’s material is transported to a dynamic separator, separating the fine part (cement) from the coarse part (see Figure 1).

Due to the transport in open wagons, the additives used for grinding, slag, ash, and limestone require drying up to a humidity of 4.5%. The cement is taken over by a transport relay and stored in the cement silos, the coarse part returning to the mill for a new crushing. The cement is then taken up and transported to the silos, either with a conveyor belt and elevator or with a system of pneumatic gutters and elevators [39].

The experimental determinations were generally aimed at finding the levels of cement dust, respectively PM_10_ concentrations, in the stacks of the cement manufacturing sector mentioned above, in two consecutive years (2019–2020). The values found were not associated with rates of illness of workers in the cement factory or the inhabitants of neighboring areas but are intended to be brought to the attention of readers, especially since the sector in which the authors work is engineering.

**Figure 3:**
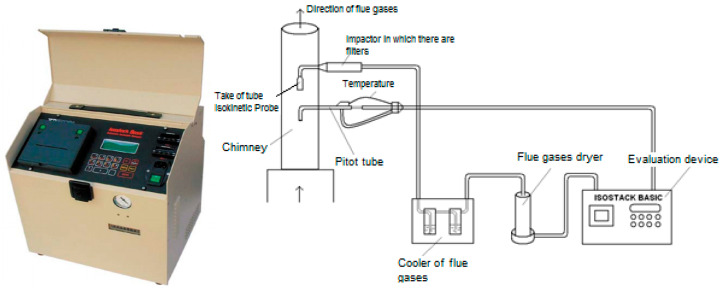
ISOSTACK BASIC HV automatic lifter and assembly diagram [40].

Monthly discontinuous monitoring of dust emissions was performed by measurements using an ISOSTACK BASIC HV automatic sampler (Tecora, Italy) (Figure 3). When installing the sampling equipment, it was taken into account that, by varying specific parameters, the particular requirements of the measurement method and the sampling location were met. In addition, before and after sampling, the tightness of the sampling line was checked.

The measurement procedure also includes performing at least one zero test, i.e., performing a measurement under the same sampling conditions as for the standard sample series, without aspirating residual gas. The zero test sample is analyzed in the same way as the samples were taken. The powder measurement method is based on the isokinetic sampling of a volume of gas from the waste gas stream, the deposition of particles on a filter element (stainless steel cartridge filled with quartz wool), and the gravimetric measurement at an analytical balance according to SR EN 13284-1: 2018 [41]. The gas sample is aspirated using a sampling probe mounted in the flue gas duct against the flow of the waste gas flow. Condensation of water vapor from the gas volume was avoided by mounting a silica gel drying tower before the filter cartridge.

Extractive sampling for the collection of dust, carried out isokinetically, means that the waste gas’ velocity at the sampling point must be equal to the suction rate of the sample [35]. An inadequate sampling rate can result in separation phenomena that eventually lead to overestimating or underestimating the particle concentration. The sampling period was 60 min (1 cartridge every 30 min), according to SR ISO 9096: 2017 [42]. The measured and calculated values, measurement methods, and operating conditions of the mill (stack conditions of temperature, pressure, humidity, etc.) were recorded in the test report and then processed. The Isostack Basic continuously measures the speed of the waste gas and adjusts the suction speed automatically. Other data collection and analysis procedures were PS-004-LM, PS-005-LM (CEPROCIM), SR EN 15259: 2008, SR ISO 14164: 2008, SR EN 14790: 2017, and SR EN 16911-1: 2013. The baskets’ height at both measuring points was 30 m, but the diameter of the two stacks was different: at the filter at the mill outlet, the basket had a diameter of 1680 mm, and at the exit of the separator, the stack had a diameter of 1260 mm.

Factory emissions are expressed as concentrations, i.e., the mass of the substance emitted in relation to the volume of emitted gas (e.g., mg/m^3^). The emissions indicated as concentrations refer to the volume of gas discharged under normal conditions (0 °C and 1013 mbar), but these must be converted to basic dry gas, which means relative to the volume of gas after subtracting the moisture content. The measured emissions of the concentrations must finally be converted to the oxygen reference value of 10% vol (dry). Local (national) emissions are expressed in the same way as those emitted at the factory, but the standard conditions (standard temperature TS, standard pressure pS) are TS = 20 °C (or 25 °C, in some situations), and pS = 1013 mbar.

The equipment used to determine the amount of dust must be calibrated using comparative measurements with an extractive method (e.g., EN 13284-1-4), and by establishing the analysis function, the concentration under the stack conditions is calculated. Calibration must be performed at least once a year by an official third-party supplier.

$$c_{dust}\left(\mathrm{mg}/m^{3}\right)=A\cdot x+B$$

where *x* is the signal from the dust equipment (mA, mg/m^3^, etc.) and *A*, *B* are the calibration factors.

The volume (m^3^) refers to the conditions in the stack (pressure, temperature, and humidity) and must be converted to the “normal conditions” of the factory (1013 mbar, 0 °C, dry gas) by the following relation:

$$c_{dust.wet}\left(\frac{\mathrm{mg}}{m_{N.wet}^{3}}\right)=\frac{T_{stack}\left(K\right)}{273\left(K\right)}\cdot \frac{1013\left(\mathrm{mbar}\right)}{p_{stack}\left(\mathrm{mbar}\right)}\cdot c_{dust}\left(\frac{\mathrm{mg}}{m^{3}}\right)$$

where *T_stack_* is the measured absolute temperature (K) and *p_stack_* is the measured absolute pressure (mbar).

In general, the stack pressure is variable but not measured, considering a constant value of 970 mbar and the average barometric pressure value. Next, the conversion from wet to dry material is done using the following relation:

$$c_{dust.dry}\left(\frac{\mathrm{mg}}{m_{N.dry}^{3}}\right)=c_{dust.wet}\left(\frac{\mathrm{mg}}{m_{N.wet}^{3}}\right)\cdot \frac{1}{1-\frac{H_{2}O\;\left(\%\mathrm{vol}\right)}{100}}$$

where H_2_O (%, vol.) is the moisture content of the wet powder.

The correction to the factory reference standard for an oxygen content of 10 (%, vol) must be calculated with the oxygen content of the dry powder and not with that of the wet powder.

$$c_{dust.factory}\left(\frac{\mathrm{mg}}{m_{N}^{3}}\right)=c_{dust.dry}\left(\frac{\mathrm{mg}}{m_{N.dry}^{3}}\right)\cdot \frac{21-10\left(\%\mathrm{vol}\right)}{21-O_{2}\left(\%\mathrm{vol}\right)}$$

For the conversion of the values from the factory reference standard (1.01325 bar, 0 °C, dry, 10% vol O_2_) to the “local standard” (1.01325 bar, *t_s_* °C, dry, *O_2__/ref_* %vol), all values at the level factory *c_dust.factory_*, can be multiplied by the so-called country factor *f_c_*, calculated by the relation:

$$f_{c}=\left(\frac{273}{273+t_{s}}\right)\cdot \frac{21-O_{2/ref}\left(\%\mathrm{vol}\right)}{11}$$

then:

$$c_{dust.local}=f_{c}\cdot c_{dust.factory}$$

For the experimental results presented in the paper, the dust concentration in the installation gases, *C_dryed_*, was determined by reporting the obtained dust mass to the volume of pipeline extracted gas, *V_dryed_*:

$$C_{\mathrm{dryed}}=\frac{Dust\;mass}{V_{dryed}}=\frac{Final\;mass-Initial\;mass}{V_{dryed}}\;\left(\frac{\mathrm{mg}}{m_{N}^{3}}\right)$$

The values recorded at the stacks in the cement manufacturing section at an existing factory in Romania, which was permanently modernized in the last 10–15 years, were taken into account so that the total dust emissions and their components fall within regulated limits, both at a national and European level. Thus, the keywords for this paper are cement factory, cement dust, PM_10_, air pollution, and maximum permitted limits.

## 3. Results and Discussion

The main measurements were performed between March and December 2019–2020 at a cement factory in Romania at measurement points A and B (Figure 1): the filter at the outlet of the cement mill (point A) and the filter at the outlet of the mill separator (point B). The aim was to determine the average monthly concentration (mg/Nm^3^) from the two points concerning the maximum limit of the integrated environmental permit; the AIM of the cement plant. The measurements were performed under reference conditions for atmospheric emissions: 273 K, *p* = 101.3 kPa, dry gas, 10% O_2_ concentration [43].

To start the measurements, the reference O_2_ = 21% and the humidity in the pipe, measured with a multifunctional device TESTO 400 Flue Gas Analyzer (Keison products, Kirchzarten, Germany), must be entered in the ISOSTACK BASIC (TCR Tecora SRL, Via delle Primule, 16,Cogliate (MB), Italy). The device then automatically calculates all the indicators necessary to determine the concentration of dust (flow, speed, temperature, and dynamic pressure in the pipe, as well as the volume of wet and dry gas extracted in the set time—30 min/cartridge).

At the exits of the mill, before the emission stacks, two dust filters are provided, a bag filter FS76, which connects directly to the cement mill (before point A—Figure 1), and another bag filter, indicative of FS 7-21, which relates to the cement mill separator (before point B). Some experimental results obtained for the dust concentration, such as for PM_10_, at the cement mill stack, are presented in Table 1.

The measurements performed in working conditions at the cement factory gate registered the following values:

- In April, PM_10_ values were about 35 mg/m^3^ in the air, both in 2019 and 2020;
- In June, the PM_10_ values were 27.68 mg/m^3^ in 2019 and 29.26 mg/m^3^ in 2020.

Similar values of PM_10_ in the air at the factory gate were also obtained in September, respectively, 28.82 mg/m^3^ in 2019 and 26.12 mg/m^3^ in 2020, the determinations being recorded for 24 h.

Next, the distribution of the concentrations obtained during the whole measurement period at the two measurement points were represented: cement mill no.1 (MC1) of the factory—filter outlet mill and separator filter outlet (point A and point B—Figure 1).

**Figure 4:**
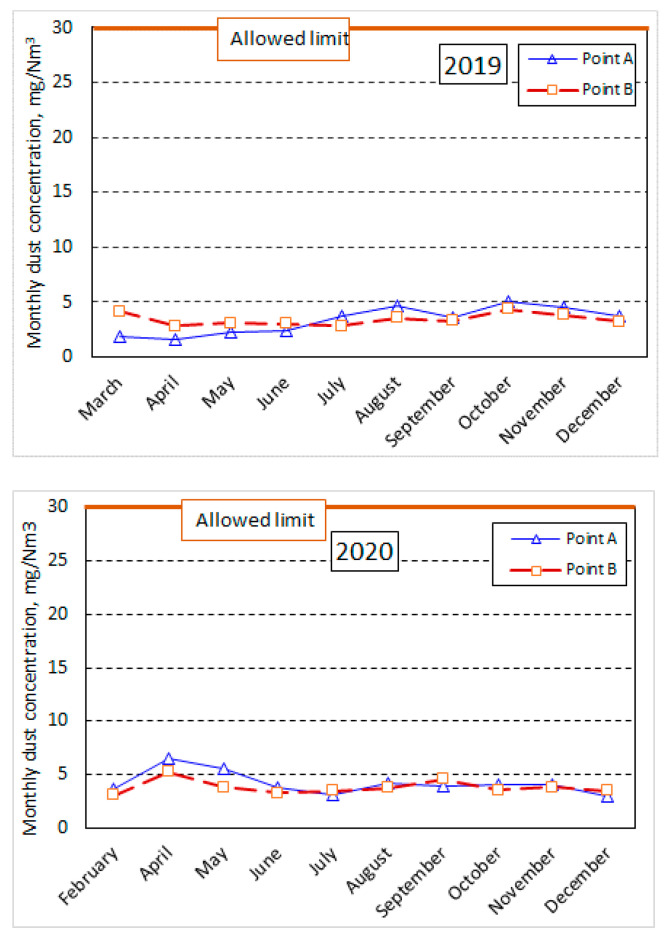
Distribution of dust emissions at bag filters in the cement mill section no.1 (mill filter—point A and separator filter—point B, Figure 1) of the analyzed cement plant.

Generally, the mean values recorded on the sampling days and hours in the months listed in Table 1 were within the prescribed normal limits (30 mg/Nm^3^), with minor variations from one month to the next (as shown in Figure 4). However, higher values of dust emissions at other hours of the factory’s work schedule or other days of the month in which samples were taken were not excluded.

The results of Soussia et al. [15] showed that the average values of cement powder inside the cement plant were higher in January and December (up to 8.752 g/m^2^/day in January and up to 9.362 g/m^2^/day in December) and lower in the summer months (up to 5.2502 g/m^2^/day in August). Additionally, the highest values of dust particles deposited on surfaces outside the factory were also in the winter months (up to 17.731 g/m^2^/day in January and up to 18.962 g/m^2^/day in December).

In our determinations, the average dust concentrations at the “Cement Mill” working point were within limits provided by the Integrated Environmental Authorization (30 mg/Nm^3^ for each working point). The presented values are the average of two consecutive determinations for 30 min each. Thus, in March 2019, the sum of the dust concentration at the two sampling points mentioned in the paper was about 6 mg/Nm^3^, well below the value of the dust concentration recorded at the working point “Coal Mill”, which was 24.57 mg/Nm^3^, but also below the level registered at the “Grill Cooler” electro filter of the factory. Overall, the dust concentration, recorded together at the two sampling points mentioned (Figure 1), was about 10.5% of the total dust concentration recorded at all dust emission points. It was found that the monthly average recorded at the level of dust concentrations was about 6764 mg/Nm^3^, together at the two sampling points, for 2019 and 7.98 mg/Nm^3^ for 2020, with a weight approximately equal in the year 2019 and slightly lower in 2020 for the sampling point “Mill separator” (i.e., 3.8 mg/Nm^3^ at the separator of the mill—point B, compared to 4.16 mg/Nm^3^ for the stack of the cement mill—point A). Thus, the dust concentration level increased by about 18% in 2020 compared to 2019. We do not know the reasons, but we can make some observations regarding:

− The level of cement production, which also increased compared to the previous year;
− The degree of cleaning of the elements of bag filters or electrofilters that need to be cleaned/replaced periodically.

However, we have some information showing that the type of cement manufactured at the time of the measurements was different (manufacturing recipe).

Regarding the average dust concentration levels in the colder months compared to the warmer months, these values were calculated separately for mid-year (May–October) compared to the other months mentioned in Table 1 (for the beginning and end of the year). Although we did not notice any significant variations in these concentrations, we could say that in 2020 these concentrations were about 4% higher in the colder months than the warmer months of the year. The phenomenon was precisely the opposite of the level of 2019. However, we can note that the months of 2019 with a slightly higher level of dust concentration in the stack of the cement mill were August, with 4.6 mg/Nm^3^, October, with 5.06 mg/Nm^3^, and November, with 4.54 mg/Nm^3^. In 2020, August also showed a slightly higher dust concentration level (about 4.21 mg/Nm^3^), but in April, there was an even higher value (6.44 mg/Nm^3^) than the other months of the year to the stack of the cement mill. Additionally, the determinations showed a fineness of the dust collected inside the mill filter of about 3000–3100 cm^2^/g. At the separator (and annexes) filter, the dust showed a specific Blaine surface (SSB) of about 11,100–11,600 cm^2^/g. This dust was integrated into the cement mass, as suggested in Figure 1, and was not collected in the stack into the atmosphere. The temperature at the two stacks (determination points) was, on average, about 55.8 °C at the mill filter outlet (point A) and about 59.6 °C at the separator filter (point B).

Collateral, the fineness of the dust from the electrofilters of the factory dust control system was about 3598 cm^2^/g (average SSB value), and the dust from the clinker oven filter showed a residue on the sieve of 90 μm (R90μm) of about 0.77%, an average SSB fineness of about 11,200 cm^2^/g and a moisture content of 0.29%. The percentage of dust that passed through the 1 μm sieve was about 16.1%, while through the 4 μm sieve passed a percentage of 62.4%, with a particle size value of about 2.8 μm at over 50% of the mass dust from the clinker oven filter.

In the case of the dust collected at the cement mill filter, the R90_μm_ residue showed an average value of 3.93%, and R64_μm_ was about 11.92%, while the dust from the separator filter showed an R90_μm_ residue of about 0.2% and an average R64 Rm residue of 0.66%, which means that most of the dust particles have dimensions below these values.

One cement-type manufacturing recipe was 75.89% clinker, 11.33% limestone, 8.66% gypsum, and 4.12% kiln filter dust. Other recipes were tested in the factory’s specialized laboratory.

We must mention that the dust was carried by atmospheric currents and deposited on the ground in different quantities depending on the distance from the factory. The total concentrations of dust, PM_10_, and PM_2.5_ exceeded the values set by the standards near the cement plant at a distance of less than 300 m, as shown in [44]. Al Smadi et al. found that, at 250 m from the factory, the dust concentration in the air was 359.61 μg/Nm^3^, while at a distance of 500 m, the ambient dust concentration was 214.58 μg/Nm^3^ (measurements of 24 h in 5 consecutive days in March 2008).

**Figure 5:**
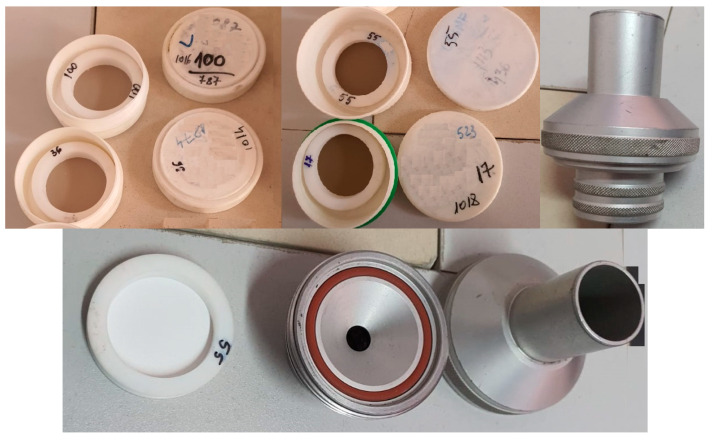
Filters after PM_10_ measurements.

Regarding the concentration of particulate matter, we only determined the PM_10_ concentration at the factory gate under working conditions, using the specific procedures provided by law, i.e., PS-005-LM and SR EN 12341: 2014, using an Echo PM installation (Tecora, Italy) and a TESTO 400 (Kirchzarten, Germany) device for measuring airspeed and ambient air quality (Figure 5). The measurements for PM_10_ took place over 3 months each year from 2018–2020, and the atmospheric conditions and the results obtained are mentioned in Table 2 (taking into account the mention in Table 1, according to which the values were well below the regulated limit).

When performing an MS Excel statistic of the data presented in Table 1, we found that the average monthly values recorded at the cement mill stack in 2019 were 3.35 mg/Nm^3^ total powders, with a standard deviation of about 1.17 mg/Nm^3^ and an arithmetic mean of the absolute deviations of the points from their average value of 1.05 mg/Nm^3^. Additionally, for a confidence interval IC = 95%, the deviation from the average value of the monthly dust concentrations was ±0.72 mg/Nm^3^.

Proceeding in the same way and considering the values presented in Table 2 for PM_10_, we could conclude that their average monthly value, under normal conditions, for the 3 years in which this parameter was monitored, was about 31.62 µg/Nm^3^, with a standard deviation of about 4.07 µg/Nm^3^ and an arithmetic mean of the absolute deviations of the points from their average value of 3.90 µg/Nm^3^. Thus, for a confidence range, IC = 95%, the deviations from the mean value of monthly concentrations of PM10 was ±2.66 µg/Nm^3^.

Although Sahri et al. [45] states that the highest concentration of PM_10_ was obtained in the autumn measurements (64.92 ± 3.76 µg/m^3^), and the lowest concentrations of PM_10_ were measured in spring and summer, our measurements show that the highest PM_10_ average concentrations were mainly during the spring. Still, they were below the legal limit provided by the AIM (50 μg/m^3^). However, the order was maintained because no determinations were made in the middle of autumn or in winter. Monthly measurements could likely lead to statements such as those of Sahri et al. [45] or Leone et al. [46], who came to approximately the same conclusion, that the highest concentrations of PM_10_ are obtained in winter, followed, in order, by the concentrations during spring and autumn and the lowest during summer (these have been confirmed by our measurements).

However, it is interesting what Sanchez-Soberon states [13,47], that meteorological conditions have an important influence on the level of concentrations of material particles in the atmosphere and environment, which would require the continuation of such determinations. The wind speed was not high during our measurements, but Sanchez-Soberon [13,48] and other researchers claim (which is pertinent) that the wind is the carrier of particles and the smaller the particle is, the easier it is to carry them further from the source. Furthermore, Sanchez-Soberon [48] determined that approximately 60% of the PM_10_ mass collected near a cement plant comprises PM1 regardless of the season. The fine fractions, such as PM_2.5_ and PM_1_, are present in a higher quantity in most toxic substances, such as some heavy metals (As, Pb, Cd, Mn, Cr) and HAPs. Correspondingly, health problems are more significant after exposure to PM_2.5_ than PM_10_ and have a more negative impact when PM_1_ is present.

Ahmad et al. [48] also showed that PM_10_ and PM_2.5_ concentrations vary in different units in the cement industry in Hattar, Haripur. The highest average concentrations of PM_10_ and PM_2.5_ were 1552 and 7867.5 (µg/m^3^), identified in the factory’s main crusher and, respectively, at the cement mill.

Following the measurements presented in the paper, several conclusions and solutions can be drawn to reduce the pollutants emitted in the cement factories in Romania:

- By installing a third generation O’Sepa separator (Henan Zhengzhou Mining Machinery Co. Ltd., Jiangsu, China), the mill’s specific consumption can be reduced by improving the recovery of coarse material, and the separation efficiency is better than with conventional separators with cyclone batteries.
- Moreover, there is a very high possibility of increasing the grinding plant’s production by 10–30% and the possibility of mechanical adjustment.

All previous studies analyzed and summarized by the authors present the risk of exposure of workers in cement plants and residents in the vicinity of the factory to dust particles, especially in terms of lung and respiratory functions, regardless of whether or not they are smokers. In addition, there are also associated with various diseases depending on the concentration of PM_10_ and PM_2.5_ in total particulate matter and on the concentrations of heavy metals, which affect the organelles of the human body (heart, liver, spleen, bones, muscles, etc.).

No determinations have been made regarding the concentration of heavy metals in the dust evacuated to the emission chimneys considered in the paper. However, we consider their determinations, mainly in the chimney of the clinker furnace, to be the main source of heavy metal emissions in the cement factory. We will return soon to the results of such determinations, as they have already been started.

## 4. Conclusions

This paper is a case study of a Portland cement plant in Romania, the experimental determinations presented mainly focusing on dust emissions in an important sector of the plant (cement mill and related facilities), with dust extraction from two chimneys in working areas (cement mill filtration system and grinder component separator filtration system), as well as the concentration of PM_10_ in this air with dust evacuated to the chimneys. According to the monitoring protocol, the recordings were made within the permanent monitoring service of the factory’s emissions through an authorized monitoring center. The results of the determinations were not correlated with the impact of dust and PM_10_ emissions on factory workers or the population in the area of the factory, nor on the fauna and flora of the area, except by comparison with the results of other researchers presented in various published papers, the recorded data being presented as such.

The average annual concentration achieved (mg/Nm^3^) in 2019 for dust emissions into the atmosphere was approximately ~10% of the maximum limit of the operating license (of 30 mg/Nm^3^), that is 3.35 mg/Nm^3^, at the emission point FS64 the bag filter of the cement mill, and 3.41 mg/Nm^3^ at FS7-21 the bag filter of the cement mill separator.

It can be said that the average monthly values for the dust discharged to the stack of the cement mill evacuation system from the technological scheme of the monitored factory fall within limits, these being in 2019, approximately 3349 ± 1164 mg/Nm^3^, below the maximum limit of the operating authorization. The dust content discharged into the atmospheric air at the stack of the mill separator exhaust system also showed values below the regulated maximum limit, which had average values of 4.18 ± 1.02 mg/Nm^3^, with a standard deviation of ±0.74 mg/Nm^3^. Deviations from the average value of monthly dust concentrations in this basket showed ±0.63 mg/Nm^3^ for a confidence interval of 95%.

The PM_10_ values, registered at the factory gate, were within the limits of 31.62 ± 3.90 µg/Nm^3^, with a standard deviation of about ±4.07 µg/Nm^3^. For a confidence interval IC = 95%, deviations from the mean value of monthly concentrations of PM_10_ were ±2.66 µg/Nm^3^.

Note that well-planned management of dust control options (installing dust collectors and/or bag filters where needed, periodic inspections, isolation of dust production operations—grinding components, screening grinders, and mixing cement components) can reduce dust generation insignificantly, with low additional costs.

In any case, the factory management and operational management’s sustained efforts over the last 10 years had success and considerably reduced dust emissions into the atmosphere, well below the limits set by the regulations and in the AIM.

## Figures and Tables

**Table 1:** The results of the measurements regarding the dust emissions at the two stacks of the cement mill MC1 of the cement factory for 2019.

No.	Period	Emission Source	Mean Monthly Dust Concentration *, mg/Nm^3^		PM10 (μg/m^3^) in Air, 2019/2020	Max Limit in AIM, mg/Nm^3^
			2019	2020		
1	March 2019/ February 2020	Cement mill MC1	1.87	3.61 **		* 30 mg/Nm^3^, for average monthly dust concentration ** 50 μg/Nm^3^, for PM10 in atmospheric air
		MC1 mill separator	4.13	3.03 **		
2	April	Cement mill MC1	1.59	6.44	32.82/35.09	
		MC1 mill separator	2.81	5.24		
3	May	Cement mill MC1	2.29	5.6		
		MC1 mill separator	3.08	3.83		
4	June	Cement mill MC1	2.39	3.83	24.80/29.26	
		MC1 mill separator	3.02	3.29		
5	July	Cement mill MC1	3.73	3.06		
		MC1 mill separator	2.83	3.46		
6	August	Cement mill MC1	4.60	4.21		
		MC1 mill separator	3.58	3.74		
7	September	Cement mill MC1	3.63	3.91	24.52/26.12	
		MC1 mill separator	3.27	4.56		
8	October	Cement mill MC1	5.06	4.06		
		MC1 mill separator	4.36	3.55		
9	November	Cement mill MC1	4.54	4.11		
		MC1 mill separator	3.80	3.81		
10	December	Cement mill MC1	3.79	2.97		
		MC1 mill separator	3.24	3.49		

* Measurements in working conditions at the gate of the cement plant in normal conditions (0 °C, 1.013 bar). ** The 2020 measurements were conducted at the end of February.

**Table 2:** The measured data for PM10 were transformed for standard temperature and atmospheric pressure (0 °C and 1013 bar) for 3 months in 2018, 2019, and 2020.

Period	Temperature *, °C	Atmospheric Pressure *, kPa	Relative Air Humidity *, %	Wind Speed *, m/s	PM_10_ in Work Conditions **, μg/m^3^	Extensive Relative Uncertainty (%)	PM_10_ in Normal Conditions **, μg/Nm^3^	Extensive Relative Uncertainty ***, (%)
17/18 April 2018	13.7/11.5	95.81/96.46	71.5/73.6	0.09/0.09	33.18	±12	37.70	±9
8/9 August 2018	21.5/20.0	96.23/96.76	50.6/52.3	0.7/0.2	25.32	±20	28.71	±15
3/4 October 2018	12.3/8.5	94.48/95.50	72.3/71.6	0.18/0.23	32.17	±11	36.25	±10
27/28 March 2019	10.5/10.3	97.06/97.46	46.3/47.1	0.6/0.6	32.82	±12	34.98	±12
13/14 May 2019	15.4/16.5	95.70/96.68	59.1/57.7	0.2/0.18	24.80	±17	27.68	±14
21/22 August 2019	21.5/22.7	96.55/95.47	48.7/47.5	0.19/0.2	24.52	±18	28.82	±15
1/2 April 2020	7.9/9.1	97.05/97.19	64.7/63.4	0.2/0.18	32.70	±12	35.09	±11
15/16 June 2020	21.7/20.9	99.7/100.25	68.5/69.8	0.21/0.21	25.31	±17	29.26	±10
1/2 September 2020	17.8/18.5	95.11/95.46	62.8/61.5	0.8/0.8	24.51	±18	26.12	±17

* The values were determined at the beginning and the end of the 24 h sample. ** The values were mediated for the 24 h duration of the sample. *** Extended uncertainty k = 2 at a 95% confidence level (k—coverage factor).
